# Immigration policy as public health policy: Trump’s first administration and a critical public health response to the second

**DOI:** 10.3389/fpubh.2025.1517287

**Published:** 2025-04-14

**Authors:** Claudia M. Calhoon, Sirry M. Alang

**Affiliations:** ^1^Graduate School of Public Health and Policy, City University of New York, New York, NY, United States; ^2^Health and Human Development, University of Pittsburgh, Pittsburgh, PA, United States

**Keywords:** immigration, racism, equity, immigration policy, critical public health

## Abstract

Although President Trump was unable to pass new immigration laws in his first administration, he successfully employed executive orders, rulemaking, and administrative practices that altered significant policies, including benefits programs, immigration enforcement, and criteria for admissibility to the United States. The public health consequences of immigration policies under Trump’s first presidency, deeply punitive immigration policies at the forefront of his 2024 presidential campaign, and far-reaching executive orders that have characterized the early days of his second term present important challenges to practitioners. Arriving at a consensus about how public health institutions respond to political health determinants has become more urgent as increasingly restrictive and punitive immigration policies are proposed and implemented. Critical perspectives that explore the role of power in policy development, implementation, and impact, such as critical race theory, can help public health practitioners do this. We discuss the 45th administration’s immigration policies in terms of colorblindness and race-consciousness, whiteness and entitlement, interest convergence, and the ubiquity of racism. We show how these policies extend dangerously into the current 47th presidency. The impacts of restrictive and punitive immigration policies, as well as the continuous desensitization of the public to extreme approaches, require critical public health responses.

## Introduction

Although President Trump was unable to pass new immigration laws in his first administration, he was successful in employing executive orders, rulemaking, and administrative practices that transformed life for immigrants in the United States (1–6). Exposure to policies in the 45th administration has been associated with increases in preterm births (7, 8), heightened cardiovascular risk (9), reduced health care utilization (10), and diminished self-reported health status among immigrants and their families (11). In addition, under former president Trump, the nation experienced gaping racial and ethnic disparities in COVID-19 mortality (12) and a 150% increase in anti-Asian trauma and injury-causing hate crimes reported to the police in the 18 largest U.S. cities (13, 14).

During the 2024 election campaign, the president’s lies about Haitian immigrants in communities like Springfield, Ohio (15) showcased the campaign’s willingness to use immigrants as vehicles to advance deeply anti-democratic changes in the rule of U.S. law. The public health consequences of immigration policies under Trump’s first presidency and a flurry of immigration-related executive orders since January 2025 present important challenges to public health practitioners and policymakers. Immigration policy is now typically understood within public health to be a social determinant of health but what that means in practice is less clearly defined (16, 17). Articulating that is increasingly more urgent as successively more restrictive policies become normalized in our political dialog. Critical perspectives that explore the role of power in policy development, implementation, and impact, such as critical race theory, can help public health practitioners do this.

### Critical race theory

Critical race theory (CRT) is a set of tools, narratives, and analytical approaches to show how laws sustain structural racism (18–20). CRT seeks to uncover how laws promote racial disparities and discrimination, especially laws related to immigration that on paper appear neutral or colorblind (21). Instances of racial discrimination and inequality are not aberrations from an otherwise just system that successfully guarantees equality under the law (21). Many laws are designed with the explicit intention of exploiting a workforce or maintaining the racial *status quo.* But even laws that appear to be neutral can sustain inequality.

### Immigration policy in the 45th administration and prologue to the 47th

Although former president Trump did not invent the U.S. immigration system, his administration weaponized it in unique ways among recent presidential administrations (3, 22). Shortly after entering office in 2016, Trump issued executive orders that summarily rendered 11 million undocumented people immediate priorities for removal from the country (3, 23). President Trump’s ban on travelers from seven majority-Muslim countries was implemented over a weekend, leaving travelers with visas and green cards stranded worldwide (24). His public charge rule changed immigrant use of public benefit programs, heightened distress, and altered their relationship with community service providers (25). Even efforts considered unsuccessful from a policy perspective contributed to a documented “chilling effect” inhibiting immigrants from health care and public benefits for which they were qualified (25–28). A partial list of first administration actions is in Table 1.

**Table 1:** Selected first-term trump immigration policy activities (1, 3, 78).

January 1, 2021 status	Date	Agency	Outcome
Implemented	January 2017	Department of Homeland Security	Executive order on deportation prioritizing every unauthorized immigrant for removal
Implemented	January 2017	Department of Homeland Security	Increased enforcement activities on buses and trains within 100 miles of US borders
Implemented	January 2017	Department of Homeland Security	Ban on 7 majority Muslim countries
Stopped	January 2017	Department of Justice	Withholding of federal funds from cities or states that decline collaboration with ICE
Pending	May 2017	Department of Defense	Barriers to naturalization of foreign nationals in the US military
Implemented	June 2017	Depts. of State and Homeland Security	Slowing of responses to visa and green card applications
Stopped	September 2017	Department of Homeland Security	Rescission of deferred action of childhood arrivals (DACA)
Pending	September 2017	Department of Homeland Security	Ending of temporary protected status for 6 countries
Implemented	January 2018	Department of State	State Dept. public charge screening
Implemented	January 2018	Department of State	Empowerment of department officers to limit the period for which nonimmigrant visas are valid
Stopped	April 2018	Depts. of Justice and Homeland Security	Blanket criminal prosecutions for all who enter the US unlawfully (Zero Tolerance)
Stopped	March 2018	Commerce Department	Placement of untested question about citizenship on the 2020 Census
Implemented	August 2018	Department of Homeland Security	Expanding criteria for being considered public charge/ineligible for residency
Pending	September 2018	Department of Justice	Rule parallel to DHS rule to determine circumstances in which someone could be deported for public charge status
Implemented	January 2019	Department of Homeland Security	Migrant Protection Protocols (MPP), requiring migrants, mainly asylum seekers, to wait in Mexico for their adjudications
Pending	February 2019	Social Security Administration	Proposed rule making lack of English proficiency no longer a factor for eligibility for Social Security disability insurance
Pending	July 2019	Department of Homeland Security	Regulation making migrants ineligible for asylum if they failed to apply for it elsewhere *en route* to the United States
Implemented	September 2019	Department of Homeland Security	Asylum Cooperation Agreements with Central American countries allowing US to send asylum seekers abroad
Pending	November 2019	Department of State	Proclamation stating that all new immigrants may be denied entry unless they show eligible health insurance or have sufficient resources for foreseeable medical costs
Implemented	January 2020	Department of State	Lowest refugee admissions ceiling since creation of modern US refugee resettlement program in 1980
Implemented	February 2020	Department of Homeland Security	Heightened immigration enforcement in cities that limit cooperation with ICE
Implemented	March 2020	Centers for Disease Control	Order mandating that all foreign nationals without authorization to enter US be pushed back to Mexico (or Canada) or returned to their countries
Implemented	May 2020	Depts. of Agriculture, Health and Human Services, and Social Security	Proclamation ordering agencies to begin enforcing financial commitments of immigrant sponsors
Pending	Ongoing	Department of Defense	Funding for border wall: as of Jul 2020, Trump had administration obtained $15 billion for wall, although only 30% was appropriated by Congress
Pending	May 2019	Department Housing and Urban Development	HUD mixed-status rule to prevent unauthorized immigrants from living in subsidized housing

Past is prologue. In the first six weeks of the 47th administration, Trump has already issued a series of executive orders addressing both immigration and race. They range from mass deportations to halting refugee resettlement programs, ending processes to seek asylum at the border, and expanding the power of ICE to carry out raids in areas previously deemed sensitive such as churches, hospitals, and schools (29). He also spurred mass dismissals of civil servants across multiple agencies, destabilizing institutions and populations that depend on them. A partial list of second administration actions to date is in Table 2. These actions impact public health outcomes and practice and require a response from researchers and practitioners to mitigate harm. We discuss these through the lens of CRT.

**Table 2:** Selected second-term Trump immigration policy activities (29, 78).

February 28, 2025 status	Date	Agency	Outcome (EO # if applicable)
Implemented	January 20, 2025	Census Bureau	Rescinded Biden Executive Order (EO) affirming apportionment of Congressional representation based on all inhabitants of the state regardless of immigration status (14148)
Implemented	January 20, 2025	Depts. of State, Justice, Homeland Security, Offices of Management and Budget and Personnel Management	Rescinded Biden rule rescinding first Trump administration guidance on immigration enforcement and sanctuary cities (14148)
Implemented	January 20, 2025	Depts. of State and Homeland Security	Rescinded Biden rule addresses regional causes of migration in the Americas (14148)
Implemented	January 20, 2025	Depts. of State, Justice, and Homeland Security	Rescinded Biden Rule strengthening immigrant integration and inclusion (14148)
Implemented	January 20, 2025	Depts. of State, Department of Homeland Security	Rescinded Biden Rule addressing refugee resettlement (14148)
Pending	January 20, 2025	Depts. of State, Justice, Homeland Security, and Social Security Administration	Issued Executive Order saying that 14^th^ amendment does not extend citizenship universally to everyone in born within the United States (14160)
Implemented	January 20, 2025	Depts. of State, Justice, and Homeland Security	Required additional vetting and scrutiny for visa approval processes for hostile attitudes toward the US or support threats to national security (14161)
Pending	January 20, 2025	Department of Homeland Security	Suspended the US Refugee Admission Program (14163)
Pending	January 20, 2025	Depts. of Justice, Homeland Security and Defense	Resumed erecting physical barriers on Southern border (14165)
Pending	January 20, 2025	Depts. of Justice, Homeland Security and Defense	Deployed additional personnel (14165)
Pending	January 20, 2025	Depts. of Justice, Homeland Security and Defense	Ended “catch and release” practices (14165)
Pending	January 20, 2025	Depts. of Justice, Homeland Security and Defense	Resumed Migrant Protection Protocols (14165)
Pending	January 20, 2025	Depts. of Justice, Homeland Security and Defense	Promoted federal-state collaboration on immigration enforcement (14165)
Pending	January 20, 2025	Depts. of Justice, Homeland Security and Defense	Altered parole policies including terminating the CBP One application (14165)
Implemented	January 20, 2025	Department of Defense	Ordered deployment of US military to the southern border (14167)
Pending	January 21, 2025	Department of Homeland Security	Ended “Sensitive Locations” policy banning immigration enforcement in schools, hospitals, and houses of worship
Implemented	January 29, 2025	Depts. of Justice, Homeland Security and Defense	Expands scope of immigration domestic immigration enforcement’s ability to deport undocumented individuals accused of crimes
Pending	January 30, 2025	Department of Homeland Security	Terminated 2023 Temporary Protective Status (TPS) for Venezuelans
Pending	February 1, 2025	Depts. of State, Justice, and Homeland Security	Expanded scope of national emergency at Southern border to the Northern border with Canada, threatening tariffs (14193)
Pending	February 1, 2025	Depts. of State, Justice, and Homeland Security	Threatened Mexico with tariffs in case of failure to stem migration and drug traffic (14194)
Implemented	February 4, 2025	Department of Homeland Security	First flight with detainees sent to Guantanamo Bay
Implemented	February 7, 2025	Depts. of State and Homeland Security	Promoted resettlement of Afrikaner refugees fleeing “racially discriminatory property confiscation” (14204)
Implemented	February 19, 2025	Depts. Justice, and Homeland Security	Directed agencies to identify any programs that may be providing public benefits to undocumented immigrants. (14218)
Pending	February 24, 2025	Department of Homeland Security	Reduced extension of TPS for Haitians

### CRT and Trump’s immigration policies

#### Colorblindness and race-consciousness

CRT highlights the need for vigilance in identifying emergent mechanisms of discrimination (30). CRT describes colorblindness as a declared absence of racial thinking in perceptions, attitudes, and practices (31). Policies implemented in a nominally color-blind manner foster structural racism by allowing an unspoken preference for whiteness (20). CRT theorists have described immigration policy as a “technology of racism” that gives proponents of restrictive policies non-racist-sounding language to employ when proposing constraints on the dignity, mobility, and safety of non-white populations (32). Technologies of racism allow the mechanisms that privilege whiteness to shift and reconstitute themselves, creating the illusion of progress while sustaining a status quo in which racialized legal statuses restrict movement, benefits, access to employment, or services (30).

Although President Trump used racist comments and innuendo throughout his first presidency, he simultaneously claimed not to “see color,” to fend off criticism over racially charged language (33, 34). In one instance, he complained about Democratic Congressional representatives Rashida Tlaib (birthplace, Michigan), Alexandria Ocasio-Cortez (birthplace, New York), Ayanna Pressley (birthplace, Ohio), and Ilhan Omar (raised in the U.S. and now a citizen), saying “Why do not they go back and help fix the totally broken and crime infested places from which they came? …” (35). In response to the ensuing uproar, his campaign invoked colorblindness to provide him plausible deniability for the use of derogatory language. A Trump campaign official declared: “Donald Trump does not see color. He does not see race” (36, 37). But even while making this disclaimer, the president suggested that these elected officials were not qualified to participate in government (36). In his 2024 presidential run, Trump dispensed with any plausible deniability, referring to his Black female opponent as low-IQ and immigrants crossing the Southern border as “animals” (38).

For over half a century, the U.S. has been referred to as a nation of immigrants—celebrating how diversity and contributions of people from other countries and ethnicities strengthen the U.S. But once he returned to office, President Trump issued an executive order that banned efforts to improve recruitment and retention of employees across race or ethnic groups (39). In the wake of the American Eagle Flight 5,342 air traffic accident on January 28, 2025, he attributed responsibility for the deaths to the efforts to ensure a diverse applicant pool, telling White House pool reporters, “they [Obama Administration] actually came out with a directive, too white” (40).

#### Whiteness and entitlement

CRT argues that whiteness bestows entitlement and functions as a resource (41). Race, as a social construction, is sustained by the acceptance of whiteness as the normative default and standard against which other groups are defined (42). In 1790, the first US immigration law designed only “free, white” persons as eligible to apply for naturalization as citizens (42). Rooted in the need to distinguish an individual subject to enslavement from an individual who was not, the status of whiteness “became a shield from slavery.” (41) [p. 1720]. Trump’s immediately-enjoined executive order purporting to end “birthright citizenship” is entitled “Protecting the Meaning and Value of American Citizenship” (35). Attempts to end birthright citizenship and eliminate diversity, equity, and inclusion devalue the lives and contributions of racially minoritized populations, many of whom are immigrants. They also minimize the legacies of systemic oppression and exclusion, and privilege whiteness across all sectors and institutions.

Whiteness has continued to shape the distribution of social and economic benefits. The Trump administration pursued a variety of ways to penalize immigrants who used public benefits that impoverished white Americans are perceived to be exclusively entitled to (43, 44). The U.S. Department of Homeland Security (DHS) *Rule on Inadmissibility on Public Charge Grounds* (45–47) expanded the list of benefit programs in which participation could prompt adverse immigration consequences to include Medicaid, food stamps, and any type of federally financed housing benefit (45). Heightened scrutiny was designed to activate Trump supporters’ fears that immigrants were using resources meant for them (41).

These policies obscure immigrant contributions in the labor market and tax base. Even as immigrants contributed $492.4 billion federally in 2019 (48), the rule suggested that people recently admitted to the U.S. could not “rely on their own capabilities.” (45) Even though most of the penalized benefit programs were already unavailable to individuals without a green card, the policy reducing participation in the use of WIC, SNAP, and health insurance among immigrants in all status groups (27). As many as 260,000 children may have been unenrolled in Medicaid because of the rule (26). In his second administration, an Executive Order issued on February 19, 2025, revived the rhetoric of the 45th administration public charge rule, ordering all federal agencies to “identify all federally funded programs administered by the agency that currently permit illegal aliens to obtain any cash or non-cash public benefit ….,” implying that while everyone contributes to the economy, not everyone is entitled to benefit from the same (49).

#### Differential racialization

How policies affect one racialized population relative to another is frequently driven by labor needs, political power, foreign policy considerations, or a combination of the three. CRT observes that each non-white racialized group has its own unique set of adverse experiences with structural racism (42). Often these experiences manifest in instances where, as one group sees progress, more blatant attacks are trained on another (42). Differentially racialized groups may find it difficult to act in solidarity with one another when they are conscious of the variations of their narratives in the U.S. These tensions are easily exploited by those in power to prevent solidarity. Trump’s reliance on the specter of external threats to the U.S., or the “enemy at the gates,” (50) appeared designed to create a siege mentality among his supporters while also deepening differential racialization of U.S. residents (51). Trump’s executive order *Protecting the Nation from Foreign Terrorist Entry*, known as the Muslim ban, racialized Muslims as outsiders and as a danger, similar to what happened after September 11, 2001. Linking a religion to terrorism reinforces a racialization of all adherents of that religion, contributing to interpersonal and community-level tensions (52).

Despite World Health Organization recommendations against naming newly emerged diseases for a particular geographic area, Trump referred to COVID-19 as the “China Flu” and “Kung Flu” numerous times as the pandemic propagated (53). Twenty-four tweets by President Trump with stigmatizing language implicating China or Chinese individuals were retweeted 1,213,700 times for a total of 4,276,200 likes between January 1 and August 31, 2020 (53, 54). Trump’s response to COVID-19 coincided with a dramatic increase in attacks on Asian Americans (55). Between 2019 and 2020, although overall reported hate crimes diminished by 7%, incidents targeting Asians increased by 150%. Between March 2020 and the end of the year, 4,193 hate crimes and incidents were documented by the organization Stop AAPI Hate (55).

#### Interest convergence and the ubiquity of racism

“Interest convergence” describes how progress in racial equity is limited to moments in which the majority white population’s interests converge with the demands of communities of color, or when there is a perceived win-win situation. When these interests no longer align, progress may be undone or blocked. Derrick Bell proposed this concept in the 1970s, noting that major civil rights successes of the 1960s were only achieved when the interests of white-dominated power structures converged with the demands of communities of color (30). Specifically, at the time of the 1954 *Brown v. Board of Education* decision, it was the convergence of interests between Black demands for integration and white concerns about the United States’ Cold War image, fear of civil unrest at home, and the need for economic development in the South that allowed progress in school integration (56).

Trump’s narrative of divergent interests was prominently displayed in this attempt to remain president after he lost in 2020. While speaking to supporters protesting the certification of election results on January 6, 2021, before violently taking over the federal Capitol, he said, “If you do not fight like hell, you are not going to have a country anymore” (57). His use of the border as a political prop and rallying cry to energize his followers and as a backdrop for visible and punitive immigration enforcement actions (58) highlights how racism seeks to protect the interest of white people (32, 59). Indeed, in January 2025, he halted refugee resettlement programs and ended processes for undocumented immigrants to seek asylum at the border – continuing to reassure his supporters that the U.S. belongs only to them and that immigrants are bad for the country (60).

CRT itself was targeted by President Trump. In September 2020, he issued an executive order prohibiting federal agencies and their contractors from including anything that could be construed as CRT in employee diversity training (61–63). Opponents of inclusion and anti-racism initiatives in universities, schools, companies, and local governments have changed their target from a largely fictional presence of CRT to Diversity, Equity, and Inclusion (DEI) programs and initiatives (64). As of January 2025, bills restricting how teachers or state agencies may teach or train about racism have been introduced in 44 states, and 18 states have enacted bans (65). Immediately upon entering the White House, declared that federal hiring had come to reflect a “commitment to illegal racial discrimination under the guise of equity” (66) the administration moved to memorialize these restrictions federally with executive orders that banned DEI initiatives within federal agencies and (67) directed federal agencies to spur private employer bans (39).

## Discussion

### A critical public health response

The degree to which a single presidential administration was able to shape immigration policy, as well as the detail and expansiveness of policies in Tables 1, 2, highlight the urgency of articulating critical public health responses to them. It is no coincidence that the administration’s declarations are accompanied by efforts to gut agencies tasked with caring for the health of the public. In the face of these efforts to remake public health practice, public health professionals must sharpen critiques of policies that stigmatize communities. In 2021, Alang et al. (68) proposed that the ten essential public health services (ESPHs) organized under three overarching public health functions (assessment, policy development, and assurance) must reflect an explicit focus on structural racism and white supremacy. Although EPHSs were revised to better reflect health equity, explicitly identifying how white supremacy and structural racism impact health outcomes is a part of public health practice (69). That public health agencies are currently being censored, publicly available health data restricted or deleted, and evidence-based analytical frameworks like equity and justice precipitously declared illegal highlight why an explicit focus on white supremacy is necessary to improve public health. Countering white supremacy must include articulating the public health benefits of expansive and welcoming immigration policies, as Daniel Dawes’ has said, thinking of immigration policy as a political determinant of health (70).

Young and Crookes propose that public health practitioners do this by “shifting its unit of analysis from racial/ethnic categories to the structures and systems that are the source of power, racialization, and racial inequities” (17). Ford and Airhihenbuwa (19) have urged the adoption of public health critical race praxis, an approach that incorporates CRT elements into public health research to achieve structural change. Support for research and programs to assess how racialized and discriminatory immigration policies harm the health of immigrants – and in turn overall population health, should be part of public health programming. For example, interventions to correct misinformation and explain how immigration sustains our economy (71) are part of a critical public health agenda (68, 72). Dismantling public health agencies, threatening global health diplomacy by leaving the WHO, banning collective education about slavery and its legacy, and making it illegal to consider how systems and structures harm specific populations will, in the end, harm us all.

Advocating for change at institutional and policy levels is now essential to the survival of our profession. Bassett and Graves urged public health programs and practitioners to focus on institutional practices that protect existing power configurations that adversely affect health (69). We echo their call and argue that it will become impossible to do so without strong vocal opposition to the current administration. Public health leaders must directly engage with elected officials, agencies, and organizations that are part of the public health ecosystem. We must challenge policies that stigmatize communities, assess public health harms of negative framing, and demonstrate the health value of inclusive policies. Valuable examples of this work include the New York City’s evaluation of the ActionHealthNYC pilot which laid the groundwork for its NYC Care program (73, 74). Working with a cohort of undocumented immigrants to provide referrals and schedule primary care visits, the program showed a 29% increase in primary care access and a 49% reduction in emergency visits (74). Even more powerful than city-based initiatives are state changes like California’s expansion of health coverage to all income-eligible residents for Medi-Cal (75). Better population health outcomes are possible with the implementation of inclusive policies.

Movement building and advocacy are critical public health responses for this moment. Immigrant communities led efforts to mitigate the onslaught of Trump’s first-term policies (76). Undocumented immigrants specifically increased their civic engagement over the course of the 45th administration (77). Part of public health practice is mobilizing and supporting community responses to current restrictive, stigmatizing, and punitive policymaking. Researchers and practitioners must join advocacy efforts to build political power alongside marginalized communities, challenge detrimental policies, promote inclusive practices, values, and language, and support communities harmed by this administration’s actions. The health of the most socially, economically, and politically marginalized amongst us can be a strong indicator of our values as a profession. We must act quickly and decisively.

## Conclusion

Donald Trump’s political career has prompted an unprecedented number of restrictive and punitive policies. The impacts of previous policies, public health threats of current policies, and the desensitization of the public to successively more stigmatizing policy and rhetoric will impact health outcomes and exacerbate health inequities for generations to come. Researchers and practitioners cannot treat these policies as neutral, or remain silent in the face of their implementation. CRT provides us with analytic tools and frames to advocate for structural changes that will secure health equity in the United States.

## Data Availability

The original contributions presented in the study are included in the article/supplementary material, further inquiries can be directed to the corresponding author/s.
